# Interactive Echocardiography Translation Using Few-Shot GAN Transfer Learning

**DOI:** 10.1155/2020/1487035

**Published:** 2020-03-19

**Authors:** Long Teng, ZhongLiang Fu, Qian Ma, Yu Yao, Bing Zhang, Kai Zhu, Ping Li

**Affiliations:** ^1^Chengdu Institute of Computer Application, University of Chinese Academy of Sciences, Beijing, China; ^2^Sichuan University of Media and Communications, Chengdu, China; ^3^West China Hospital, Sichuan University, Chengdu, China

## Abstract

**Background:**

Interactive echocardiography translation is an efficient educational function to master cardiac anatomy. It strengthens the student's understanding by pixel-level translation between echocardiography and theoretically sketch images. Previous research studies split it into two aspects of image segmentation and synthesis. This split makes it hard to achieve pixel-level corresponding translation. Besides, it is also challenging to leverage deep-learning-based methods in each phase where a handful of annotations are available.

**Methods:**

To address interactive translation with limited annotations, we present a two-step transfer learning approach. Firstly, we train two independent parent networks, the ultrasound to sketch (U2S) parent network and the sketch to ultrasound (S2U) parent network. U2S translation is similar to a segmentation task with sector boundary inference. Therefore, the U2S parent network is trained with the U-Net network on the public segmentation dataset of VOC2012. S2U aims at recovering ultrasound texture. So, the S2U parent network is decoder networks that generate ultrasound data from random input. After pretraining the parent networks, an encoder network is attached to the S2U parent network to translate ultrasound images into sketch images. We jointly transfer learning U2S and S2U within the CGAN framework. Results and conclusion. Quantitative and qualitative contrast from 1-shot, 5-shot, and 10-shot transfer learning show the effectiveness of the proposed algorithm. The interactive translation is achieved with few-shot transfer learning. Thus, the development of new applications from scratch is accelerated. Our few-shot transfer learning has great potential in the biomedical computer-aided image translation field, where annotation data are extremely precious.

## 1. Background

Echocardiography education has dramatically helped students to master cardiac structure assessment by combining cardiac ultrasound images with simulators. However, a more efficient method of interactive translation between ultrasound images and theoretically sketch images is still lacking. This causes the image processing difficulties in our case: echocardiography is characterized by the deformable appearance and poor spatial resolution, while limited annotations are available, building obstacles to achieve good performance as well as leverage state-of-the-art deep learning methods.

U2S and S2U are often investigated in different approaches. U2S is often specified in the segmentation task. It is addressed with the following methods: Level set (LS) [1] segmentation, Deformable templates [2, 3], Active shape models (ASM) [4, 5], Active contour methods, Active appearance models (AAM), Bottom-up approaches, and Database-guided (DB-guided) segmentation. LS and deformable templates present some drawbacks regarding the prior knowledge included in the optimization function. Active contour methods inspire the development of level set (LS) methods. ASM- and DB-guided approaches require a large number of annotated training images [6]. Bottom-up approaches are sensitive to initial conditions and lack of robustness. Additionally, none of those approaches are used to infer the sector boundary, which is essential for comprehension during education.

S2U typically models the tissue response as a collection of point scattering centers [7]. Different amplitudes are assigned to scatter from the blood pool or muscle. However, due to ignoring surrounding conditions like papillary muscles, clutter noise, and local intensity variations, the genuineness of the synthetic ultrasound images is still unsatisfactory. Some improvements in combining ultrasound recording as a template to synthetic realistic speckle textures are proposed to address the above issue [8, 9]. However, those approaches unavoidably introduced unrealistic warping in simulated speckle texture.

GAN-based translation approach recently shows its potential in generative applications [10]. Structure [11] and texture [12, 13] generation are explored in different applications. While giving an outstanding performance, the GAN approach requires sufficient annotation, which is time-consuming and expensive for biomedical applications.

**Figure 1:**
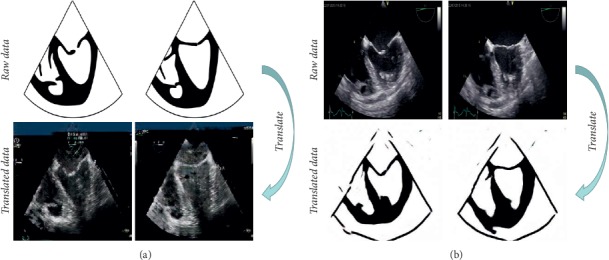
Example of interactive translation. The first two columns illustrate (a) S2U translation and the last two columns illustrate (b) U2S translation.

In this paper, we design a GAN-based transfer learning framework to interactively translate ultrasound images into sketch images (U2S translation) and sketch images into ultrasound images (S2U translation) with a handful of annotations.  Figure 1 shows the example results of final U2S translation and S2U translation.

## 2. Methods

Our approach of interactive translation consists of two steps: pretrain U2S parent network and S2U parent network and train the two networks together with end-to-end transfer learning.

Transfer learning is used for fast adaption and avoiding overfitting since we got only a handful of annotations. In our case, parent networks are carefully designed and pretrained with supervised and unsupervised learning. GAN-based few-shot transfer learning is then designed to fine-tuning the final result.

**Figure 2:**
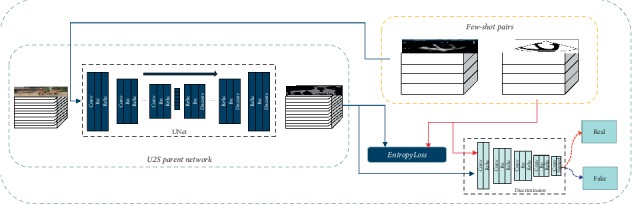
U2S network.

The proposed U2S network (Figure 2) contains a parent network that follows the U-net [14] architecture. In this paper, the U-net structure contains 10 block layers. The first five blocks are convolutional downsampling networks. Kernel size here is 3, the stride is 2, and padding is 1. Each layer is followed by a batch norm layer and a relu layer. Correspondingly, the last five layers are deconvolutional upsampling networks. Its kernel size is 4, the stride is 2, and padding is 1. The batch norm and relu layer are also adopted. Skip-connection is realized by a concatenate layer between the symmetrical layers. U2S parent network is pretrained on VOC2012 dataset [15]. During the pretraining process, the loss function is class-balanced cross-entropy.

When U2S parent network is ready, we would then transfer the U2S Parent Network into sketch translation. The Conditional Generative Adversarial Network (CGAN) [16] framework is chosen here during transfer learning to infer sector boundary. Now, the U2S Parent Network is regarded as the generation network part of CGAN. It translates ultrasound images into sketch images. The CGAN framework could intuitively generate sketch images with sector boundaries. Also, we add L1 loss as an optional criterion.

(1) $$\begin{array}{c} L_{S}=-E\left[\log\left[D_{S}\left(S,U\right)\right]\right]-E\left[\log\left[1-D_{S}\left(G_{S}\left(U\right),U\right)\right]\right]+L_{l1}. \end{array}$$

In equation (1), *D*_*S*_ is the discriminator. It contains 5 block layers. Block layers contain convolution, batch normalization, and relu layers. *D*_*S*_ determines whether the input image is translated data or ground truth. *S* represents the ground truth sketch image. *U* represents ground truth ultrasound image. *G*_*S*_ is the generator (initialized with U2S Parent Network). It translates the ultrasound image into a sketch image.

S2U recovers the ultrasound texture from the sketch. Sketch image contains only the structure and no texture information at all. We first extract and maintain texture within the parent network and then synthesis texture on the specific sketch.

**Figure 3:**
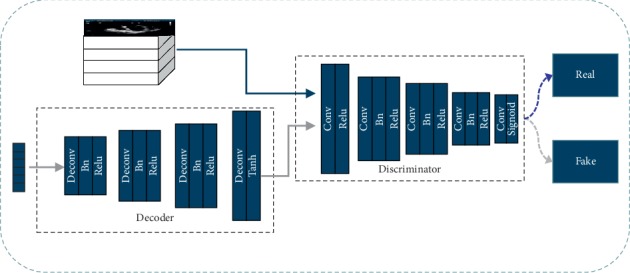
S2U Parent Network.

As shown in Figure 3, the S2U Parent Network is the decoder network. Our approach trains GAN to generate an ultrasound image on the condition of random input. In this way, as the generator part of GAN, the S2U Parent Network learns the ultrasound texture from training dataset. The S2U Parent Network consists of 4 block layers. The first 3 blocks contain a deconvolution layer, a batch normalization layer, and a relu layer. The last block contains a deconvolution layer and a tanh layer.

**Figure 4:**
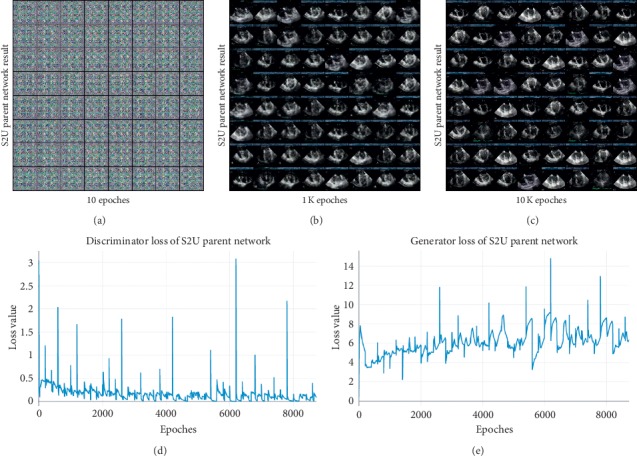
S2U training phase.

The S2U Parent Network training phase is shown in Figure 4. The generator and discriminator loss graphs are listed in the second row. The result of S2U Parent Network is illustrated in the first row. The generator and discriminator play against each other. As a result, the generator learns a growing quality of ultrasound textures.

When S2U Parent Network is ready, we could move forward to S2U transfer learning. Till now, our S2U Parent Network still has two flaws. Firstly, it cannot generate an ultrasound image on the condition of sketch input, not even pixel-level translation. Secondly, unexpected twist and image blur occur in Ultrasound Parent Network.

**Figure 5:**
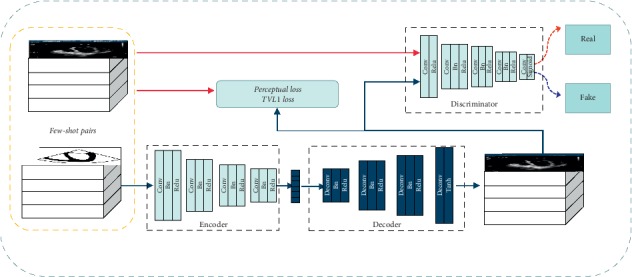
S2U network.

Aiming at making up for those two flaws, we further reform the network into S2U architecture that is shown in Figure 5. Pretrained S2U Parent Network is the dark blue part. An encoder network marked in light blue is connected to S2U Parent Network. This connection enables generation from a sketch to ultrasound image, other than from random initialization. In fact, the encoder network turns sketch image into the subset of random input. Thus, transfer learning learns the pixel-wise corresponding translation between sketch and ultrasound images. Besides, perceptual loss [17] and total variation loss are attached to the loss function. We try to maximize the fidelity of spatial resolution by minimizing GAN loss and perceptual loss. The loss function is shown in

(2) $$\begin{array}{c} L_{U}=-E\left[\log\left[D_{U}\left(U,S\right)\right]\right]-E\left[\log\left[1-D_{U}\left(G_{U}\left(S\right),S\right)\right]\right]+\lambda_{1}L_{\text{Pcpt}}+\lambda_{2}L_{\text{TV}}+\lambda_{3}L_{l_{1}}. \end{array}$$

Intuitively, the loss function of S2U is similar to equation (1). *D*_*U*_ is discriminator. It determines whether the input image is synthesized by the network, or comes from the ground truth. *D*_*U*_ has 5 block layers and is shown in Figure 5. *U* represents ultrasound ground truth. *G*_*U*_ is the generator (the combination of navy encoder and decoder). It translates a sketch image into an ultrasound image.

*L*_Pcpt_ is the perceptual loss between ground truth ultrasound image and generated ultrasound image. The perceptual loss here is calculated with the feature maps of VGG16 network, which are more invariant to changes in pixel space [18]. *L*_TV_ is the L1 smoothness of generated image. *λ*_1_, *λ*_2_, *λ*_3_ in this paper are 6*e* − 3, 2*e* − 8, and 1, which could be further optimized.

As is mentioned above, loss function in U2S and that in S2U are similar to each other. Both of them are trained under CGAN framework. Furthermore, they share the same input pairs. In Figures 2 and 4, we emphasized this similarity by marking the yellow dash blocks.

Therefore, we integrate U2S and S2U for interactive translation.

(3) $$\begin{array}{c} L_{\text{total}}=L_{S}+L_{U}. \end{array}$$

**Figure 6:**
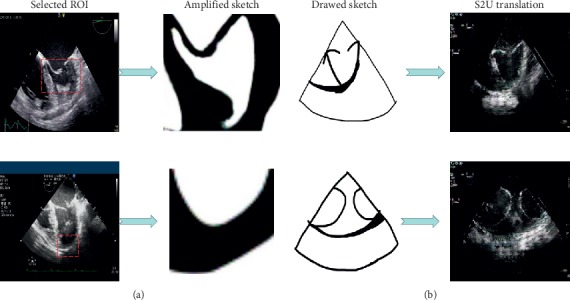
Interactive translation applications. First two columns illustrate interactive (a) U2S translation and last two columns illustrate interactive (b) S2U translation.

During transfer learning, the S2U network is trained with TVL1 loss, perceptual loss, L1 loss, and CGAN loss to maintain ultrasound texture. After transfer learning for both two networks, each network splits into the following interactive application (Figure 6 shows our applications).

### 2.1. Interactive U2S Translation

In some scenarios, the student would carefully study the static picture that captured in dynamic echo video. During this interaction, the local area should be amplified and translated into a sketch at a breakneck speed. Otherwise, the interaction would get stuck and result in a terrible experience.

In this paper, we complete sketch translation at the start of the interaction. Region of interest (ROI) is then selected and amplified to the size of the original image. Notice that, sketch image is the black-and-white image, cubic interpolation is chosen for amplification. The cubic interpolation is efficient and enough for identification.

### 2.2. Video U2S Translation

During training, automatic U2S translation would greatly help students to comprehend. Here, we split the U2S Network part from the whole networks. U2S network inputs ultrasound images and outputs sketch images. So every frame is translated into sketch images. We process frame-by-frame, converting all frames into a video. This translated sketch video is dynamically contrast to echocardiography to illustrate structural information.

### 2.3. Interactive S2U Translation

If the student draws a sketch, which outlines the cardiac structure, how the sketch corresponds to the clinical ultrasound image? This interaction could be thought-provoking and, in turn, help for comprehension.

We extract the decoder network in the S2U Parent Network and turn it into an S2U network with an encoder network. S2U inputs sketch and outputs an ultrasound image. It strictly generates output with an appropriate ultrasound texture. So, after students complete their sketch in the drawing board, the sketch image could interactively be translated into an ultrasound image.

## 3. Results

In this section, we compare the method of U2S translation and S2U translation with 1-shot, 5-shot, and 10-shot transfer learning. Firstly, the performance is analyzed through the visual comparison and the visualization of transfer learning process. Then, the performance is investigated through numerical comparison. In numerical comparison, each experiment is summarized through 45 pairs of annotations. Besides, we supplement S2U translation performance with and without perceptual loss and TVL1 loss during numerical comparison.

### 3.1. Dataset

Two datasets are used in this paper, VOC2012 and echocardiography dataset. VOC2012 is an open access segmentation dataset used for the pretraining of the U2S parent network. The echocardiography dataset is collected in the hospital under the guidance of doctors. It contains 5152 four-chamber view echocardiographs with no annotation, and 55 pairs of annotated four-chamber view echocardiographs (in this paper, we use 10 pairs for training and left 45 pairs of the annotated images for validation). Those annotations are made by the teamwork of doctors and art teachers. Images are fully annotated with the chamber (atrial and ventricular), sector boundary, and myocardial. Sensitive patient information is manually removed.

### 3.2. Visual Comparison

**Figure 7:**
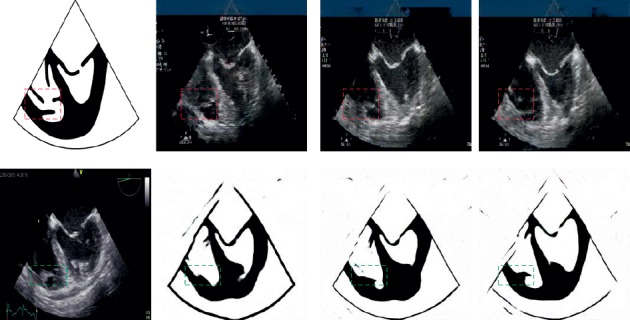
Example performance of few -shot transfer learning. The first row shows S2U of 1-shot, 5-shot, and 10-shot; the second row shows U2S of 1-shot, 5-shot, and 10-shot. The left column is a pair of ground truth.

A pair of validation images is chosen to analyze the performance of our proposed network. As shown in Figure 7, the left column is a pair of ground truth. The first row shows S2U results from 1-shot, 5-shot, and 10-shot. The contrast between myocardium and chamber is getting obvious while inputting more transfer learning data. Also, the image resolution is getting better, which makes the myocardium more realistic.

Compared with the real ultrasound images, the S2U results' texture is more similar to the training data. The blue bar and some comments from training data are synthesized on S2U results. In the second row, 1-shot, 5-shot, and 10-shot results of U2S are shown in order. The shape of the U2S result is getting similar to the ground truth. The sector boundary of U2S is also getting reasonable with more training data.

### 3.3. Transfer Learning Process

**Figure 8:**
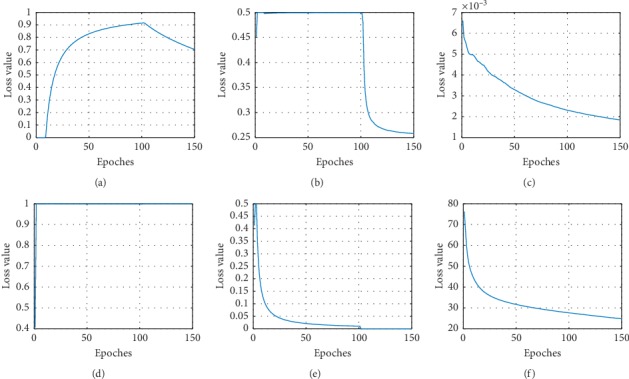
Loss function value during transfer learning. The S2U and U2S loss value during transfer learning. (a) S2U generator loss. (b) S2U discriminator loss. (c) S2U generator perceptual loss. (d) U25 generator loss. (e) U25 discriminator loss. (f) S2U generator L1 loss.

The performance of transfer learning process is investigated in two aspects, the loss function value and the corresponding performance during training. The loss function value of S2U and U2S is a representative, shown with 5-shot in Figure 8.

**Figure 9:**
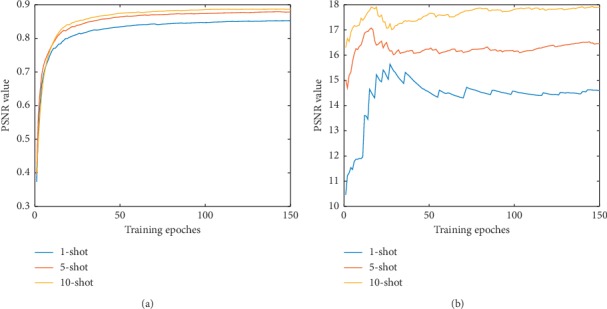
U2S and S2U performance during transfer learning. The IOU index of U2S and the PSNR index of S2U during transfer learning. (a) IOU—few-shot transfer learning. (b) PSNR—few-shot transfer learning.

As is shown in Figure 8, the first row is the first three terms of *L*_*U*_, and the second row is the terms of *L*_*S*_. The discriminator and generator loss of S2U and U2S are the first two images in the first and second rows. In both S2U and U2S, the generator and discriminator contest against each other, while the perceptual loss of S2U and the L1 loss of U2S keep decreasing. The adversarial loss function and extra loss function work together to fine-tune the final result.  Figure 9 shows the performance on testing data.

In Figure 9, the Intersection over Union (IOU) and peak signal to noise ratio (PSNR) result are representatively illustrated in 1-shot, 5-shot, and 10-shot. As a result of the proposed loss function, S2U and U2S achieve improving performance during training. Specifically, the more the training samples, the better the performance achieved. 10-shot transfer learning achieves better performance than 5-shot, while 5-shot achieves better performance than 1-shot.

### 3.4. Numerical Comparison

In U2S translation, we adopt the medical image segmentation index of dice loss, volumetric overlap error (VOE), and intersection over union (IOU). In S2U translation, we use peak signal to noise ratio (PSNR) and structural similarity index (SSIM) to evaluate our performance.

The convincing result below (Tables 1 and 2) shows the effectiveness of proposed few-shot transfer learning with 1-shot, 5-shot, and 10-shot. In Table 1, the gradual increase of training samples leads to better performance of the index. In Table 2, the indexes of PSNR and SSIM are compared with and without extra loss function.

As is shown in Table 1, few-shot learning has led to acceptable results in all of the indexes. It enables us to present the initial version of the U2S function while lacking annotations.

According to the result of Table 2, S2U that trained with the perceptual and TVL1 loss is generally better without those loss functions.

## 4. Conclusion

This paper proposed a few-shot GAN Transfer Learning for Interactive Echocardiography Translation. U2S Parent Network and S2U Parent Network are individually designed and pretrained beforehand. Then, they are assembled together for transfer learning. This joint transfer learning transfers prior knowledge into target networks. Qualitative analysis of visual comparison and visualization of the transfer learning process, quantitative analysis of numerical index shows the effectiveness of the proposed method.

The proposed method has two advantages over previous researches. Firstly, it simultaneously achieves interactive translation between ultrasound and sketch images with few-shot annotations, enabling a new educational interactive function before getting enough annotation. Secondly, it is also promising in further improvement with more training data and is promising in other related biomedical applications.

## Figures and Tables

**Table 1:** U2S translation accuracies on our dataset.

U2S	DICE	VOE	IOU
1-shot	0.902	0.001	0.852
5-shot	0.913	0.018	0.872
10-shot	0.921	−0.009	0.887

**Table 2:** S2U translation accuracies on our dataset.

S2U	PSNR	PSNR (No. perceptual and TVL1 loss)	SSIM	SSIM (No. perceptual and TVL1 loss)
1-shot	14.614	14.898	0.417	0.406
5-shot	16.180	16.074	0.499	0.433
10-shot	17.905	17.575	0.544	0.553

## Data Availability

Part of our dataset used in the current study is available from the corresponding author on a reasonable request. Our code is open source at: https://github.com/tlok666/Interactive-Echocardiograhpy-Translation-with-Few-Shot-GAN.
